# Cell-permeable transgelin-2 as a potent therapeutic for dendritic cell-based cancer immunotherapy

**DOI:** 10.1186/s13045-021-01058-6

**Published:** 2021-03-17

**Authors:** Hye-Ran Kim, Jeong-Su Park, Jin-Hwa Park, Fatima Yasmin, Chang-Hyun Kim, Se Kyu Oh, Ik-Joo Chung, Chang-Duk Jun

**Affiliations:** 1 School of Life Sciences, Gwangju Institute of Science and Technology (GIST), 123 Cheomdangwagi-ro, Gwangju, 61005 Korea; 2 Immune Synapse and Cell Therapy Research Center, Gwangju Institute of Science and Technology (GIST), Gwangju, 61005 Korea; 3 Department of Microbiology and Immunology, David H. Smith Center for Vaccine Biology and Immunology, University of Rochester Medical Center, 601 Elmwood Avenue, Box 609, Rochester, NY 14642 USA; 4 KYNOGEN Co., Suwon, 16229 Korea; 5 Department of Hematology-Oncology, Immunotherapy Innovation Center, Chonnam National University Medical School, Hwasun, 58128 Korea

**Keywords:** Transgelin-2, T-cell priming, Migration, Immunological synapse, Cell-permeable recombinant protein, Dendritic cell-based cancer therapy, Vaccine

## Abstract

**Background:**

Transgelin-2 is a 22 kDa actin-binding protein that has been proposed to act as an oncogenic factor, capable of contributing to tumorigenesis in a wide range of human malignancies. However, little is known whether this tiny protein also plays an important role in immunity, thereby keeping body from the cancer development and metastasis. Here, we investigated the functions of transgelin-2 in dendritic cell (DC) immunity. Further, we investigated whether the non-viral transduction of cell-permeable transgelin-2 peptide potentially enhance DC-based cancer immunotherapy.

**Methods:**

To understand the functions of transgelin-2 in DCs, we utilized bone marrow-derived DCs (BMDCs) purified from transgelin-2 knockout (*Tagln2*^*−/−*^) mice. To observe the dynamic cellular mechanism of transgelin-2, we utilized confocal microscopy and flow cytometry. To monitor DC migration and cognate T–DC interaction in vivo, we used intravital two-photon microscopy. For the solid and metastasis tumor models, OVA^+^ B16F10 melanoma were inoculated into the C57BL/6 mice via intravenously (i.v.) and subcutaneously (s.c.), respectively. *OTI TCR* T cells were used for the adoptive transfer experiments. Cell-permeable, de-ubiquitinated recombinant transgelin-2 was purified from *Escherichia coli* and applied for DC-based adoptive immunotherapy.

**Results:**

We found that transgelin-2 is remarkably expressed in BMDCs during maturation and lipopolysaccharide activation, suggesting that this protein plays a role in DC-based immunity. Although *Tagln2*^*−/−*^ BMDCs exhibited no changes in maturation, they showed significant defects in their abilities to home to draining lymph nodes (LNs) and prime T cells to produce antigen-specific T cell clones, and these changes were associated with a failure to suppress tumor growth and metastasis of OVA^+^ B16F10 melanoma cells in mice. *Tagln2*^*−/−*^ BMDCs had defects in filopodia-like membrane protrusion and podosome formation due to the attenuation of the signals that modulate actin remodeling in vitro and formed short, unstable contacts with cognate CD4^+^ T cells in vivo. Strikingly, non-viral transduction of cell-permeable, de-ubiquitinated recombinant transgelin-2 potentiated DC functions to suppress tumor growth and metastasis.

**Conclusion:**

This work demonstrates that transgelin-2 is an essential protein for both cancer and immunity. Therefore, transgelin-2 can act as a double-edged sword depending on how we apply this protein to cancer therapy. Engineering and clinical application of this protein may unveil a new era in DC-based cancer immunotherapy. Our findings indicate that cell-permeable transgelin-2 have a potential clinical value as a cancer immunotherapy based on DCs.

**Supplementary Information:**

The online version contains supplementary material available at 10.1186/s13045-021-01058-6.

## Introduction

Dendritic cells (DCs) are professional antigen-presenting cells that survey tissues for foreign antigens [1, 2]. Following an encounter with a foreign antigen, DCs are activated in a process involving the capture and processing of the antigen, expression of lymphocyte co-stimulatory molecules, migration to lymphoid tissues like the spleen and lymph node for completion of their maturation, and secretion of cytokines to initiate the adaptive immune response [3, 4]. During maturation and migration to the lymph node, DCs undergo global rearrangements of the actin cytoskeleton, which are mediated through specific temporal and spatial actions of actin-binding or regulatory proteins [1]. Rac1/2, a small G-protein responsible for ruffling movements, is essential for the interaction between DCs and T cells [5, 6]. The formin mDia1 is essential for DC adhesion, migration, and sustained interaction with T cells [7]. Wiskott–Aldrich syndrome protein, a molecule that controls Arp2/3-dependent actin polymerization, is required for the formation of the immunological synapse (IS) and DC migration [8–10]. The cortactin HS1 is necessary for organizing the podosome array and is primarily required for directional persistence of migrating DCs [11, 12]. However, none of these actin regulators is specific for DC functions as they are ubiquitously expressed and function in most mammalian cells [1]. Thus, the discovery of a DC-specific actin regulatory protein would help us understand how DC immunity is linked to dynamic actin remodeling at a fundamental level.

Transgelin-2, a 22-kDa actin-binding protein, is one of three transgelin family members characterized by their actin cross-linking and gelling properties [13]. Although the topic is still debated, transgelin-2 has been implicated in tumorigenesis and cancer development [14]. Indeed, its upregulation is correlated with the clinical stage, tumor size, and invasion in a wide spectrum of cancers [15]. We previously found that transgelin-2 is also expressed in lymphocytes and functions to stabilize the immunological synapse, thereby enhancing T cell activation [16, 17]. It is also involved in filopodium initiation and/or elongation presumably by interfering with the interaction between the Arp2/3 complex and actin [18], which may drive the enhanced phagocytic behavior of macrophages toward invading bacteria [19]. Taken together, these results suggest that transgelin-2 is not only important for tumorigenesis and cancer progression but is also essential for immune functions.

In the present study, we observed that although transgelin-2 is not at all or only minimally expressed in immature BMDCs, it is dramatically expressed during granulocyte–macrophage colony-stimulating factor (GM-CSF)- or FMS-like tyrosine kinase 3 ligand (Flt3L)-induced maturation and lipopolysaccharide (LPS) activation. This suggests that transgelin-2 may play a role during DC maturation or DC-mediated priming of antigen-specific T cells. In support of this idea, previous reports demonstrated that the actin bundling protein fascin is induced upon DC maturation and involved in the antigen presentation activities of mature DCs [20, 21]. Why would DCs require the expression of an actin bundling protein specific to DCs? Since the main functions of DCs, which distinguish them from other cells, are to continuously capture, deliver, and process antigens and present them to T cells [22], mature DCs may require actin regulatory proteins optimized for DC functions. In addition, this suggests that these proteins are not redundant and that each may have distinctive roles for mature DC functions. Here, we investigated the subcellular localization and functions of transgelin-2 in BMDCs. Two-photon microscopy was utilized to monitor the in vivo migration of BMDCs, as well as their dynamic interaction with T cells. BMDCs with genetic ablation of *Tagln2* (*Tagln2*^−/−^) exhibited significant defects in homing to the draining lymph node and priming of antigen-specific T cells for clonal expansion and cytokine production. Surprisingly, exogenous introduction of a cell-permeable and ubiquitination site-mutated (K78R) recombinant transgelin-2 (dU-TG2P) into BMDCs significantly potentiated tumor regression in vivo, suggesting a potential use for transgelin-2 peptides in DC-mediated anticancer therapy. In summary, our findings indicate that transgelin-2 positively regulates DC-mediated adaptive immune responses.

## Results

### ***Tagln2***-knockout (***Tagln2***^***−/−***^) ***DCs do not optimally control B16F10 tumor metastasis and growth in mice***

**Fig. 1:**
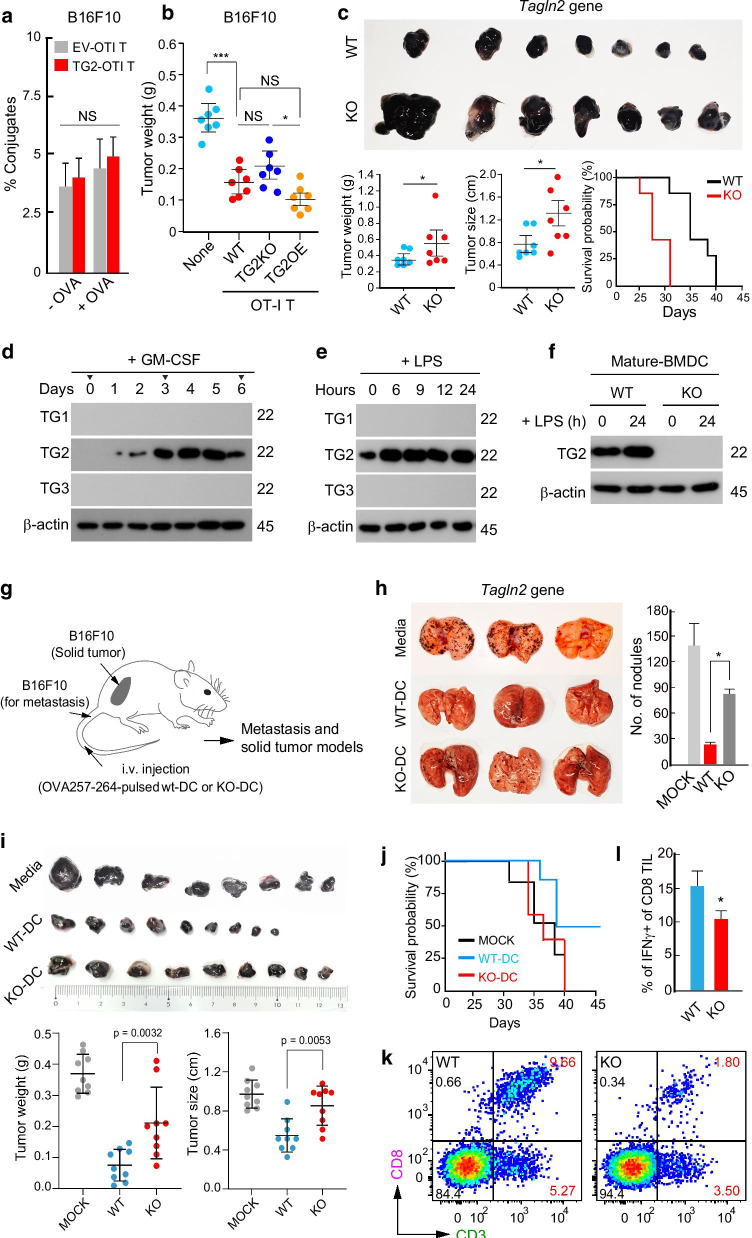
*Tagln2*^*−/−*^ BMDCs do not optimally control B16F10 tumor metastasis and growth in mice. **a** Effect of transgelin-2 expression in T cells for adhesion to B16F10 tumor cells. Empty vector (EV)- or transgelin-2 (TG2)-expressing OTI CD8^+^ T cells were co-incubated with B16F10 in the presence or absence of OVA peptide for 2 h, and conjugates were analyzed by flow cytometry. **b** Transgelin-2 in T cells showed a minimal effect on B16F10 tumor growth in mice. PBS (none), WT (OTI-T), KO (*Tagln2*^*−/−*^ OTI-T), or transgelin-2-overexpressing T (TG2OE OTI-T) cells were adoptively injected into C57BL/6 mice after OVA^+^B16F10 inoculation. B16F10 tumor weights were measured at day 25 post-implantation (n = 7). **c** Gross images of 8-day-old OVA^+^B16F10 melanoma after s.c. inoculation (3 × 10^5^) in C57BL/6 WT or *Tagln2*^*−/−*^ mice. Tumor weights and sizes were measured at day 8 post-implantation (n = 7). The survival rates of tumor-bearing mice post-implantation are shown. **d** Expression of transgelins during GM-CSF-induced differentiation. Freshly isolated BM cells were treated with 20 ng/mL GM-CSF and harvested at the indicated days for western blot. **e** Expression of transgelins in differentiated DCs after LPS (200 ng/mL) stimulation. **f** Transgelin-2 expression in DCs from WT or *Tagln2*^*−/−*^ mice. Results are representative of three independent experiments (**d**–**f**). **g** Schematic diagram of solid and metastatic tumor models. **h**, **i** Gross images of OVA^+^B16F10 lung metastasis and solid tumors. C57BL/6 mice were injected i.v. with media alone, WT DCs, or *Tagln2*^*−/−*^ DCs. After 7 days, the mice were i.v. (**h**) or s.c. (**i**) injected with OVA^+^B16F10 cells. The metastatic nodules (**h**) and tumor weights and sizes (**i**) were quantified after 8 days. **j** The survival rates of tumor-bearing mice post-implantation are shown. Data are representative for nine mice in each group. **k**, **l** Tumors from I were dissected, and the populations of infiltrated CD3^+^CD8^+^ T cells (**k**) and the amount of intracellular IFN-γ in TILs were determined by flow cytometry (**l**). All data from **a**–**l** represent the mean of three experiments ± SEM. *P < 0.05; **P < 0.001; ***P < 0.0001

Previously, we reported that transgelin-2 is highly expressed in immune-related tissues, such as the thymus, spleen, and LNs [16]. Transgelin-2 is also dominantly expressed in lymphocytes and plays an important role in stabilizing the IS, thereby enhancing T cell-mediated immune responses [16, 17, 23]. In addition, we found that transgelin-2 is physically associated with the integrin lymphocyte function-associated antigen-1 (LFA-1), which enhances the adhesion of cytotoxic T cells to intercellular adhesion molecule-1 (ICAM-1)-positive tumor target cells, such as E0771 cells, but not ICAM-1-negative B16F10 cells [17]. In accordance with the previous results, overexpression of transgelin-2 (TG2) in OTI T cells did not enhance T cell adhesion to ovalbumin (OVA)-pulsed B16F10 target cells in vitro and showed only a mild effect on B16F10 tumor regression in vivo (Fig. 1a, b). Interestingly, however, we observed that whole-body transgelin-2-KO (*Tagln2*^**−/−**^) mice, as compared to wild-type (WT) mice, developed rapid B16F10-derived tumor growth, resulting in increased tumor weights and sizes (Fig. 1c). Kaplan–Meier survival studies 40 days after tumor injection showed that WT mice had a median survival time of 35 days. However, *Tagln2*^*−/−*^ mice had a median survival time of only 28 days (Fig. 1c). These results suggest that cell types other than T cells can participate in tumor regression independently of the LFA-1/ICAM-1 interaction between cytotoxic T cells and tumor target cells.

Since DCs play a major role in processing and presenting antigen peptides to antigen-specific T cells, we next asked whether DCs express transgelin-2 or other isotypes, such as transgelin-1 and -3. Interestingly, immature DCs isolated from bone marrow (BM) did not express transgelin family members (Fig. 1d). However, transgelin-2 was highly expressed during GM-CSF-induced differentiation (Fig. 1d). The expression of transgelin-2 was also induced in fully differentiated BMDCs in response to LPS (Fig. 1e), suggesting a specific role of transgelin-2 in mature BMDCs.

To understand the significance of transgelin-2 expression in mature DCs, we used mature BMDCs from WT (*Tagln2*^+*/*+^) and *Tagln2*^*−/−*^ mice (Fig. 1f), which were previously generated in our laboratory [16]. To determine whether transgelin-2 regulates the functions of BMDCs, OVA257–264-pulsed WT or transgelin-2-knockout (KO) BMDCs were i.v. injected into mice, thereby generating cytotoxic T cell clones against the OVA257–264 peptide in vivo (Fig. 1g). At 7 days after DC injection, the mice were i.v. injected with OVA^+^B16F10 melanoma cells. The metastatic colonies were evaluated at day 14, and a significant increase in metastatic nodules was observed in mice with adoptively transferred OVA257–264-pulsed *Tagln2*^*−/−*^ BMDCs compared to OVA257–264-pulsed WT BMDCs (Fig. 1h). For the solid tumor model, OVA^+^B16F10 cells were implanted into the mammary fat pads of C57BL/6 female mice previously injected with WT BMDCs or *Tagln2*^*−/−*^ BMDCs. In accordance with the metastatic model, *Tagln2*^*−/−*^ BMDCs could not optimally control tumor growth in mice (Fig. 1i). Kaplan–Meier survival studies showed that mice injected with OVA257–264-pulsed WT BMDCs had a median survival time of 40 days. However, mice adoptively transferred with OVA257–264-pulsed *Tagln2*^*−/−*^ BMDCs had a median survival time of only 34 days similar to PBS control group (Fig. 1j). Further, we observed a significant reduction in CD8^+^ tumor-infiltrating lymphocytes (TILs) in the B16F10 tumors from *Tagln2*^*−/−*^ DC-injected mice (WT *vs.* KO, 9.8 ± 1.5 *vs.* 1.8 ± 1.1) (Fig. 1k). In addition, the tumor-infiltrating CD8^+^ T cells from *Tagln2*^*−/−*^ DC-injected mice expressed a lower level of IFNγ than that in WT DC-injected mice (Fig. 1l). Among various DC subsets, conventional type 1 DCs (cDC1s) are the main cellular source of IL-12, a fundamental cytokine for anti-cancer CD8^+^ CTL activation, and the super promising DC subset for induction of anti-tumor immunity due to their superior capacity to uptake dying or dead cell materials and to process tumor-associated antigens for cross-presentation [24–26]. We therefore determined whether transgelin-2 is also expressed in Flt3L-induced cDC1s. To this end, BM-isolated immature DCs were treated with Flt3 ligand for 9 days, and analyzed the expression of surface markers and transgelin-2 (Additional file 1: Fig. S1A and B). We found that transgelin-2 was also dramatically induced during Flt3L-induced cDC1 differentiation, while transgelin-2-KO followed the normal differentiation patterns as judged by the expressions of CD103, CD24, and XCR1 (Additional file 1: Fig. S1A and B). We next evaluated the antitumor activity of *Tagln2*^**−/−**^ cDC1s against s.c. injected OVA^+^B16F10 cells. Similar to the GM-CSF-induced BMDCs, *Tagln2*^**−/−**^ cDC1s were not able to optimally control tumor growth in mice (Additional file 1: Fig. S1C), suggesting that transgelin-2 is also important for the function of cDC1s to facilitate optimal antigen presentation.

### Transgelin-2 is essential for global actin rearrangements in activated DCs

**Fig. 2:**
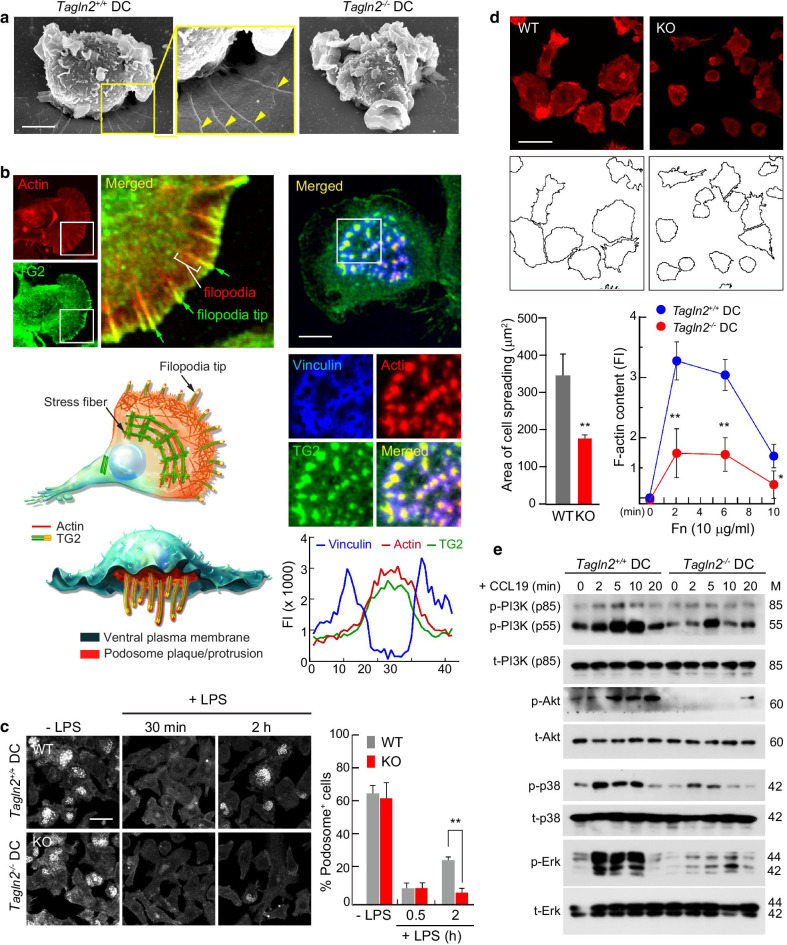
Transgelin-2 is essential for global actin rearrangements in activating DCs. **a** SEM images of WT or *Tagln2*^*−/−*^ DCs in the resting state. **b** Localization of transgelin-2 in DCs. Enlarged boxed images show the localization of transgelin-2 in the filopodial tip at the edge of a protrusive region (left) and podosomes, along with F-actin and vinculin (right). The fluorescence intensity profiles of each protein were analyzed using Fluoview. Scale bar, 5 μm. **c** Re-assembly of podosomes in WT or *Tagln2*^*−/−*^ DCs in response to LPS. Podosome cores were visualized by phalloidin staining, and the number of podosomes per cell was counted as described in Materials and Methods. Scale bar, 5 μm. **d** Effects of transgelin-2-deficiency on DC spreading and actin polymerization. WT or *Tagln2*^*−/−*^ DCs were seeded on Fn-coated coverslips and stained with phalloidin-TRITC. Cell spreading areas were calculated using ImageJ software. F-actin contents were analyzed by flow cytometry. Data from **c** and **d** represent the mean of three experiments ± SEM. *P < 0.05, **P < 0.001. **e** Determination of signaling cascade in WT or *Tagln2*^*−/−*^ DCs in response to CCL19 (200 ng/mL) by western blot. Results are representative of three independent experiments

To investigate the mechanism by which transgelin-2 affects the function of DCs, we analyzed mature BMDCs obtained from *Tagln2*^*−/−*^ mice and compared them with WT BMDCs in terms of their morphology, actin dynamics, and signaling. Morphologically, although there were no gross differences between WT and KO mice, *Tagln2*^*−/−*^ BMDCs exhibited an absence of filopodia-like membrane protrusions, as determined by scanning electron microscopy (SEM) (Fig. 2a, yellow arrowheads). To understand this morphological phenotype, LPS-stimulated WT BMDCs were stained for F-actin and transgelin-2 and visualized by confocal microscopy. As shown in Fig. 2b, transgelin-2 was specifically localized at the leading edge of lamellipodia, where filopodia are formed from the pre-existing meshwork of Arp2/3 complex-branched filaments. In addition, transgelin-2 co-localized with F-actin in the actin-rich cores of podosomes, which were surrounded by vinculin in the stereotypical podosome organization (Fig. 2b, right). Podosomes are characteristics of cells in the myeloid lineage, including DCs, macrophages, and osteoclasts, which can degrade extracellular matrices and play a role in the migration of DCs through tissues [27]. Thus, the localization of transgelin-2 in filopodia tips, as well as in podosomes, demonstrated that transgelin-2 is involved in the membrane protrusions that support DC migration and interactions with T cells for proper antigen presentation. To this end, we next assessed podosome formation and spreading during DC activation in response to LPS and fibronectin (Fn), respectively.

DCs have been shown to exhibit transient podosome loss at approximately 20 min after their activation with LPS and then recover them again after 2 h [27, 28]. Likewise, in our study, BMDCs from both WT and KO mice showed a substantial reduction in the number of cells with podosomes after 30 min of stimulation with LPS. However, podosomes were significantly slower to reform in *Tagln2*^*−/−*^ BMDCs after 2 h (Fig. 2c). Next, since the interaction of BMDCs with extracellular matrix (ECM) proteins, such as Fn, induces a dynamic cytoskeletal rearrangement driven by actin polymerization, thereby mediating cell adhesion and migration through integrins [28, 29], we evaluated the effects of the transgelin-2 KO on DC spreading and actin polymerization. Compared to WT BMDCs, *Tagln2*^*−/−*^ BMDCs exhibited reduced cell spreading and lower levels of polymerized actin when seeded on Fn (Fig. 2d). Taken together, these results strongly suggest that transgelin-2 has a specific role in DC migration by modulating filopodia and podosome formation, and cell spreading on ECM.

CCR7 is necessary for directing DCs to secondary lymphoid nodes and eliciting an adaptive immune response [30, 31]. Furthermore, CCR7 induces actin rearrangements by activating Akt and Erk signaling, as well as the G_i_-dependent activation of MAPK members Erk1/2, Jnk, and p38 [32]. To determine whether transgelin-2 is linked to dynamic actin signaling in mature BMDCs, we examined signaling downstream of CCR7 activation by CCL19. PI3K and its downstream effector Akt were both significantly attenuated in *Tagln2*^−/−^ BMDCs. Similarly, p38 and Erk signaling were also reduced in these cells (Fig. 2e).

### ***Tagln2***^***−/−***^ DCs showed impaired migration into the lymph node

**Fig. 3:**
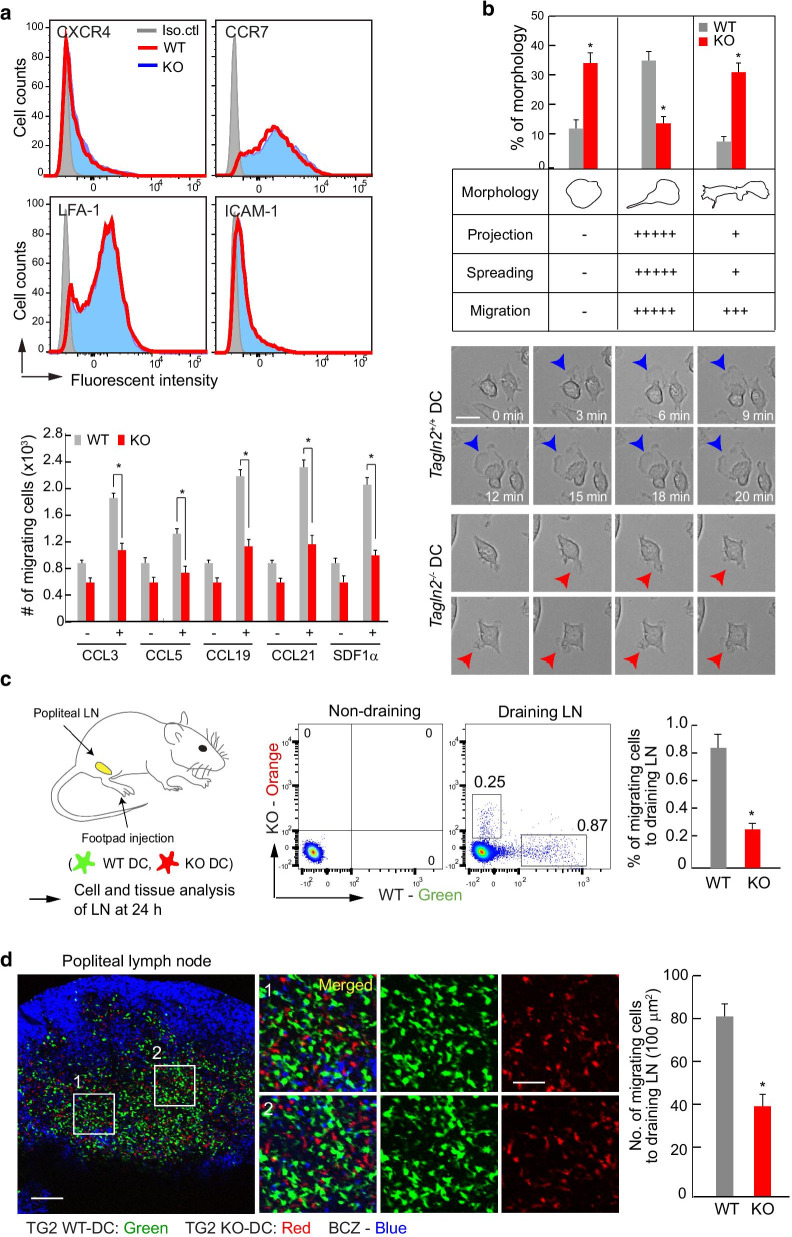
*Tagln2*^*−/−*^ DCs showed impaired migration into the lymph node. **a** Expression of chemokine receptors and adhesion molecules in WT or *Tagln2*^*−/−*^ DCs. The in vitro migration assay was performed using a Boyden chamber, and the number of migrating cells was counted by flow cytometry. **b** Time-lapse imaging of WT or *Tagln2*^*−/−*^ DCs on Fn in response to CCL19 (200 ng/mL). Representative morphology was categorized into three groups according to projection, spreading, and migration. Normal protrusions and irregular membrane morphology in WT or *Tagln2*^*−/−*^ DCs are indicated as blue and red arrows, respectively. Scale bar, 10 μm. **c** Schematic diagram of the experimental setup for **c** and **d**. **c**, **d** The same number of WT (green) or *Tagln2*^*−/−*^ DCs (red) was injected into the footpads of WT recipient mice, and the number of migratory cells in the draining popliteal LNs was analyzed by flow cytometry (**c**) and fixed cryosections (**d**). Anti-B220 was used to distinguish the B cell zone. Scale bar, 100 μm. All data represent the mean of three experiments ± SEM. *P < 0.01

Reduced CCR7-mediated signaling led us to examine whether transgelin-2 deficiency was connected with the expression of chemokine receptors or adhesion molecules on the surface of DCs. We found no differences in the expression levels of surface proteins between WT and KO BMDCs. However, *Tagln2*^−/−^ BMDCs exhibited dramatic defects in migration in response to various chemokines, such as CCL3, 5, 19, 21, and SDF-1α (Fig. 3a). We further performed live time-lapse imaging to further monitor how DCs responded to stimulation with the chemokine CCL19. Although WT BMDCs readily spread, formed membrane protrusions, and showed a typical dendritic morphology, *Tagln2*^−/−^ BMDCs were round and irregularly shaped, with a relative absence of a protrusive morphology (Fig. 3b and additional movie files show this in more detail. [See Additional files 2–5]), suggesting that the spatial and temporal regulation of the actin cytoskeleton is impaired in *Tagln2*^−/−^ BMDCs.

We therefore next asked whether DC migration was also impaired in vivo in *Tagln2*^*−/−*^ mice. To this end, we co-injected differentially labeled BMDCs from WT or *Tagln2*^−/−^ mice into the footpads of normal recipient mice. Draining popliteal LNs were collected at 24 h post-injection, and the presence of migrated DCs was assessed by flow cytometry (Fig. 3c). Fixed tissues were also examined under confocal microscopy (Fig. 3d). In contrast to WT BMDCs, which reached the draining LNs normally, there was a significant reduction in the number of *Tagln2*^−/−^ BMDCs observed in the draining LNs (Fig. 3c, d), thus demonstrating that transgelin-2 influences the migration rate of DCs in vivo.

### ***Tagln2***^***−/−***^*** DCs do not optimally support T cell activation***

**Fig. 4:**
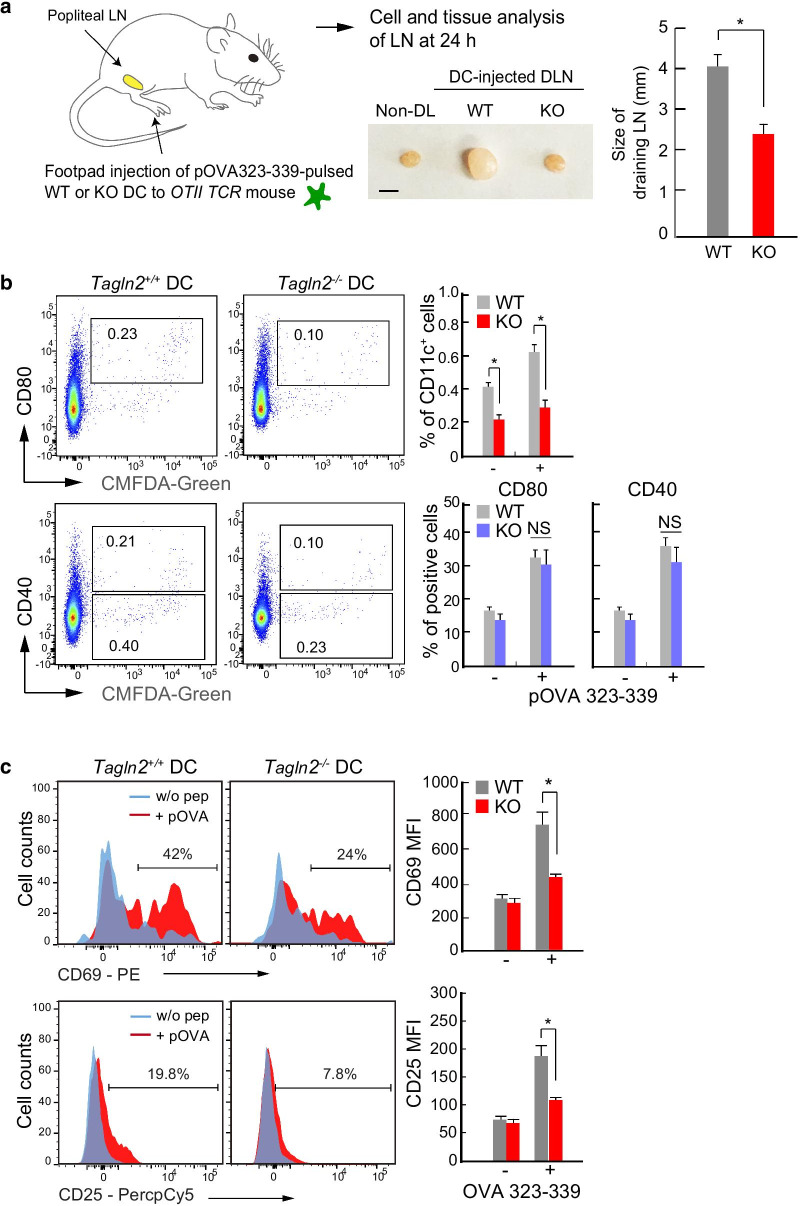
*Tagln2*^*−/−*^ DCs do not optimally support T cell activation in vivo. **a** Schematic diagram of the experimental setup for this figure (left). WT or *Tagln2*^*−/−*^ DCs were pulsed with pOVA (323–339), stained with CMFDA-green, and injected into the footpad of OTII recipient mice. A representative photograph of excised popliteal LNs (right). Scale bar, 1 μm. **b** Flow cytometric plots show activation of migrating DCs in vivo at 24 h post-injection. DCs were isolated from the popliteal LNs of recipient mice and stained with anti-CD40 or CD80. The percent of CD11c^+^ cells in the draining LNs is presented as a bar graph. **c** DC-mediated T cell activation in vivo*.* At 24 h post-injection of DCs, OTII CD4^+^ T cells were isolated from the popliteal LNs of recipient mice and stained with anti-CD69 or CD25. All data represent the mean of three experiments ± SEM. NS, not significant. *P < 0.01

Although enlarged draining popliteal LNs were observed in recipient *OTII* TCR mice adoptively transferred with OVA323–339-pulsed WT BMDCs, no significant change in LN size was seen with *Tagln2*^*−/−*^ BMDCs (Fig. 4a). This result suggests that antigen-specific OTII CD4^+^ T cells may be less proliferative in mice injected with *Tagln2*^*−/−*^ BMDCs. As expected, the population of *Tagln2*^*−/−*^ BMDCs (CD11c^+^ cells) that migrated into the draining LN was significantly smaller than that of WT BMDCs (Fig. 4b). Interestingly, however, the expression of co-stimulatory molecules, such as CD80 and CD40, was not altered in *Tagln2*^*−/−*^ BMDCs in vivo, suggesting that transgelin-2 has little effect on the ability of DCs to express these co-stimulatory factors. This result led us to ask whether the migratory defects seen in *Tagln2*^*−/−*^ BMDCs were the only driver of the reduced antitumor response (Fig. 1h, i) or whether another mechanism was also involved. To this end, we investigated the role of transgelin-2 in the direct DC-mediated T cell response in vitro. OVA323–339-pulsed BMDCs were co-cultured with OTII CD4^+^ T cells, and the surface expression of CD69 or CD25 on T cells was determined. Interestingly, the expression of these activation markers was significantly reduced in T cells co-cultured with *Tagln2*^*−/−*^ BMDCs, thereby indicating a second role for transgelin-2 in DCs.

**Fig. 5:**
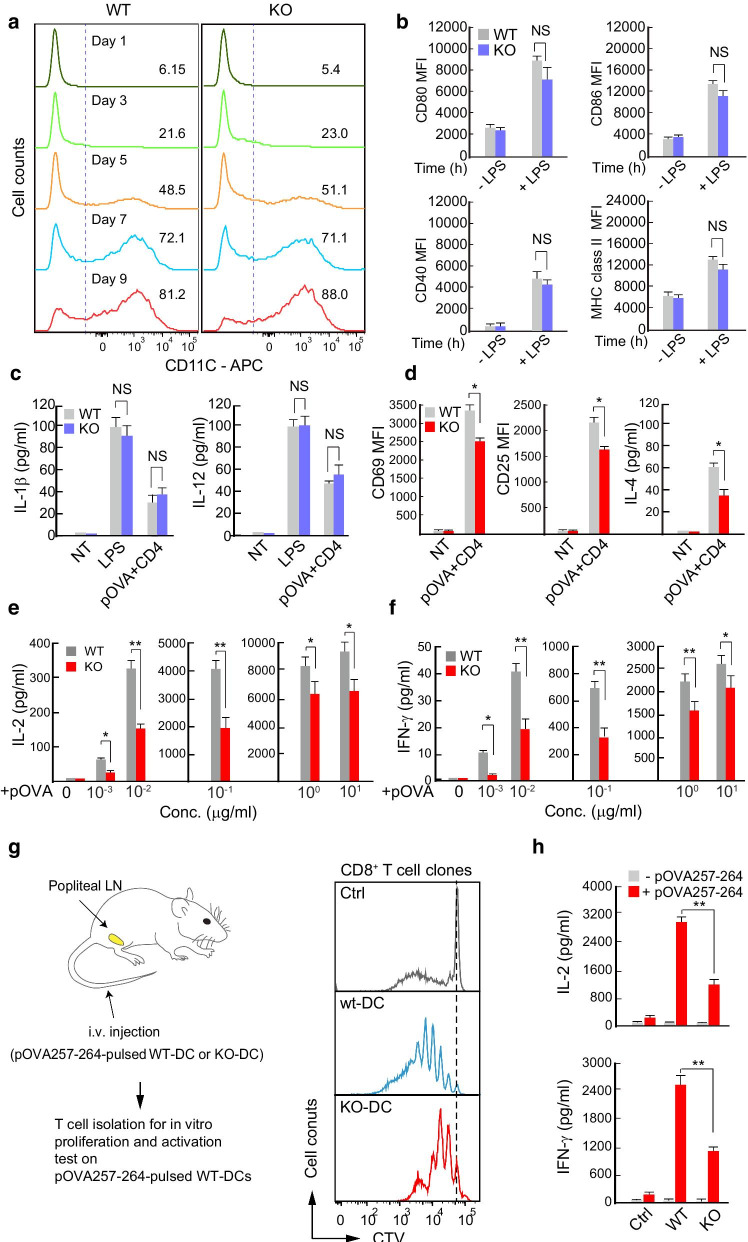
*Tagln2*^*−/−*^ DCs did not optimally support T cell activation in vitro. **a**, **c** Differentiation (**a**), activation (**b**), and cytokine secretion (**c**) of *Tagln2*^*−/−*^ BM cells. WT or *Tagln2*^*−/−*^ BM cells were cultured with GM-CSF, and then, CD11c^+^ cell populations were examined at the indicated days (**a**). Differentiated CD11c^+^ cells were further activated with LPS or pOVA (323–339), plus OTII T cells. Activation markers (**b**) and cytokine production (**c**) were determined. **d**–**f** The cells from **a** were co-incubated with OTII CD4^+^ T cells in the presence of different doses of pOVA (323–339, 10^−3^–10^1^ μg/mL), and then, T cell activation (CD69 and CD25 and IL-2, IL-4, and IFN-γ) was determined by flow cytometry and ELISA. **g** Schematic diagram of the experimental setup for G and H. Representative histogram showing the in vitro proliferation of OVA (257–264)-specific CD8^+^ T cells isolated from C57BL/6 mice administered with pOVA (257–264)-pulsed WT DCs or *Tagln2*^*−/−*^ DCs. Isolated CD3^+^ T cells were stained with CTV and co-incubated with pOVA (257–264)-pulsed WT DCs in vitro for 4 days. **h** From the supernatant of experiment **g**, IL-2 or IFN-γ production was determined at 24 h by ELISA. All data represent the mean of three experiments ± SEM. *P < 0.05; **P < 0.01

To understand how *Tagln2*^*−/−*^ BMDCs attenuated T cell activation in vivo, we performed an in vitro activation assay. We first determined whether transgelin-2 KO affected the differentiation of immature DCs into mature DCs. During GM-CSF-induced differentiation, *Tagln2*^*−/−*^ BM cells followed the normal differentiation patterns based on the surface expression of CD11c (Fig. 5a). In addition, the expression levels of MHC class II and co-stimulatory molecules, such as CD80, CD86, and CD40, were not significantly different in either WT or *Tagln2*^*−/−*^ BMDCs by LPS stimulation (Fig. 5b). Further, the expression levels of DC cytokines such as IL-1β and IL-12 were similar in both WT and *Tagln2*^*−/−*^ BMDCs (Fig. 5c). By contrast, the activation of OTII CD4^+^ T cells was significantly reduced in OVA323–339-pulsed *Tagln2*^*−/−*^ BMDCs as determined by the expression of CD69 and CD25, and the secretion of IL-4, IL-2, and IFN-γ (Fig. 5d–f). Indeed, higher levels of antigen peptide were required to produce a similar amount of cytokines in the *Tagln2*^*−/−*^ BMDCs (Fig. 5e, f). Thus, we asked whether *Tagln2*^*−/−*^ BMDCs would be unable to optimally activate antigen-specific T cells to expand in vivo. To this end, C57BL/6 mice were injected with OVA257–264-pulsed WT or *Tagln2*^*−/−*^ BMDCs via the footpad. After 7 days, CD3^+^ T cells were purified and examined for proliferation and cytokine secretion in response to the same OVA257–264-pulsed WT BMDCs in vitro. As shown in Fig. 5g, h, *Tagln2*^*−/−*^ BMDCs induced lower levels of cell proliferation and cytokine secretion, suggesting that they could not optimally support T cell clonal expansion in vivo.

### Deletion of transgelin-2 reduces DC contact with T cells

**Fig. 6:**
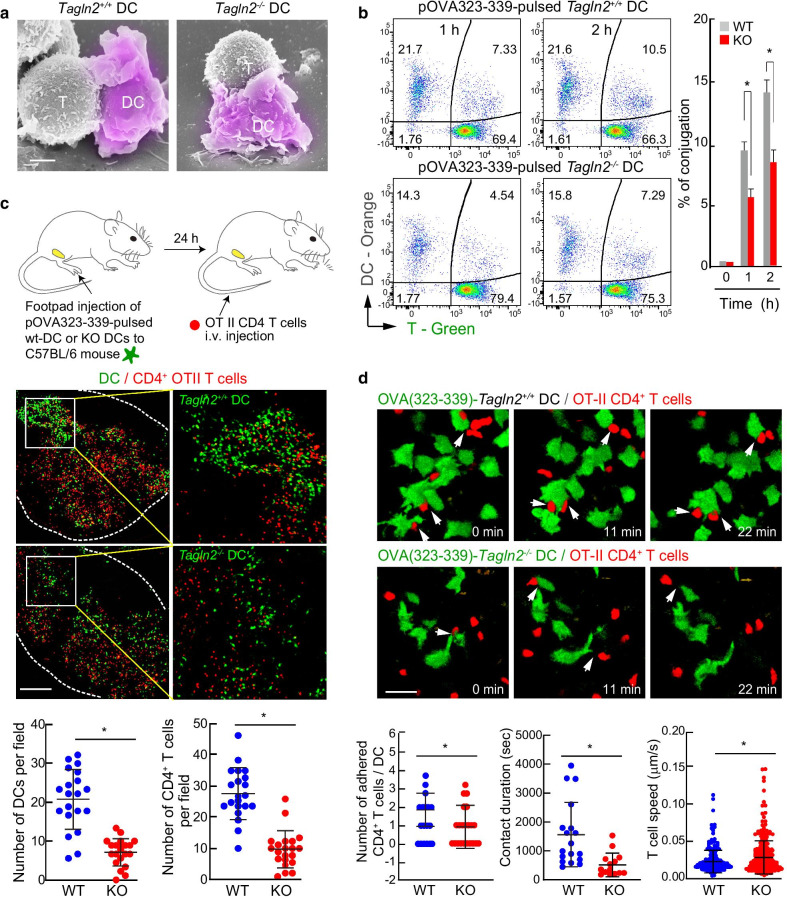
*Tagln2*^*−/−*^ DCs do not fully support T cell adhesion in vitro and in vivo. **a** SEM image of a representative DC-T cell interaction. WT or *Tagln2*^*−/−*^ DCs were pulsed with pOVA (323–339) and co-incubated with OTII CD4^+^ T cells for 1 h. Scale bar, 5 μm. **b** Reduced conjugate formation in *Tagln2*^*−/−*^ DCs. Stable conjugates were identified as double-positive events by flow cytometry. **c** Schematic diagram of the experimental setup for **c** and **d**. Representative cryosection images showing the overall distribution of WT or *Tagln2*^*−/−*^ DCs (green) and OTII CD4^+^ T cells (red) in draining lymph nodes. The statistical analysis of the number of migrated CD4^+^ T cells or DCs was performed. Scale bar, 100 μm. **d** Representative snapshot of live images of DC–T cell interactions in vivo. Draining popliteal LNs were visualized by two-photon microscopy. Scale bar, 10 μm. White arrowheads indicate the contact cells. Statistical analysis of the number of T cells in contact with one DC, the contact duration, and the T cell speed are presented. All data from **b**–**d** represent the mean of three experiments ± SEM. *P < 0.05

T cell activation and differentiation require sustained interaction with cognate DCs via integrin affinity and avidity regulation. In a previous study, we showed that transgelin-2 in T cells is associated with LFA-1 and can regulate LFA-1 avidity [17]. We therefore tested whether transgelin-2 is also involved in DC-mediated T cell adhesion. However, SEM analysis revealed no differences between WT and *Tagln2*^−*/*−^ BMDCs (Fig. 6a). To assess the adhesion strength between BMDCs and T cells in a quantitative manner, we performed a conjugation assay in the presence of an OVA peptide. We found that *Tagln2*^*−/−*^ BMDCs showed a significant reduction in conjugation (Fig. 6b), demonstrating that transgelin-2 supports adhesion between DCs and antigen-specific T cells.

To evaluate the role of transgelin-2 in regulating interactions between DCs and T cells in the live LN, we performed three-dimensional live-imaging of DCs using two-photon microscopy. OVA323–339-pulsed WT or *Tagln2*^*−/−*^ BMDCs labeled with Cell Tracker CMFDA were s.c. injected into the rear footpads of C57BL/6 mice. At 24 h post-injection, OTII CD4^+^ T cells labeled with CMRA were i.v. injected (Fig. 6c). As previously observed (Fig. 3d), a significantly smaller number of *Tagln2*^*−/−*^ BMDCs migrated into the LN as compared to WT BMDCs (Fig. 6c). Moreover, T cells formed more stable contacts with WT BMDCs than with *Tagln2*^*−/−*^ BMDCs (Fig. 6d). As a result, the T cell speed in the LN was reduced, and track displacement differences were not significant (Fig. 6d). Collectively, these results demonstrate that transgelin-2 is essential not only for the delivery of antigen materials through the dynamic movement of DCs but also for maintaining sustained interactions between DCs and T cells for the initiation of adaptive immune responses.

### Cell-permeable recombinant transgelin-2 fused with protein transduction domain (PTD) reconstitutes DC functions

**Fig. 7:**
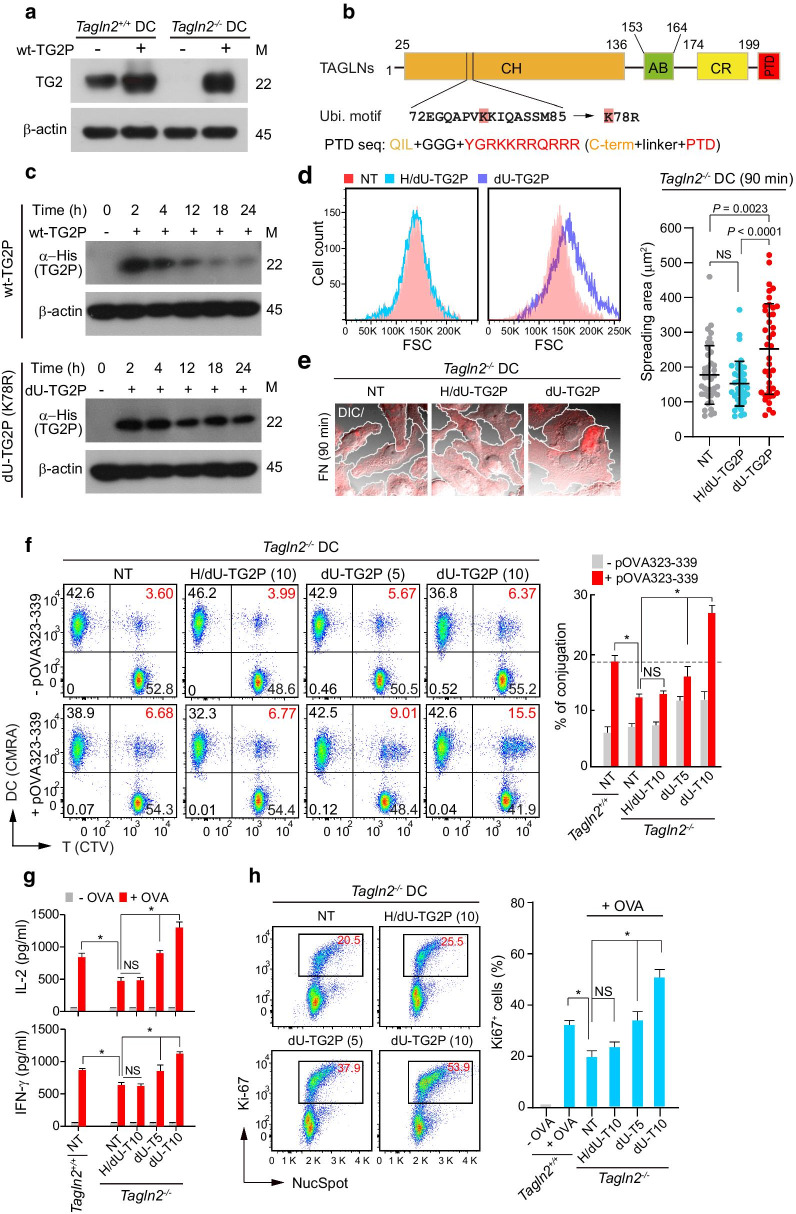
Recombinant dU-TG2P reconstitutes *Tagln2*^*−/−*^ DC functions. **a** The transduction efficiency of WT-TG2P (10 µM) in WT or *Tagln2*^*−/−*^ DCs. **b** Schematic diagram of the domain composition of transgelin-2. A potential ubiquitination site, K78, is highlighted in red. **c** Stability test of WT-TG2P and dU-TG2P (K78R) in DCs. **d**, **e** Reconstitution of transgelin-2 by dU-TG2P. *Tagln2*^*−/−*^ DCs treated with dU-TG2P or heat-inactivated dU-TG2P (H/dU-TG2P) were seeded on Fn-coated plate, and the cell size (**d**) and spreading (**e**) were determined by flow cytometry and confocal microscopy, respectively. F-actin was stained by phalloidin-TRITC (E). Cell spreading areas were calculated using ImageJ software. **f**–**h** dU-TG2P rescues transgelin-2 function. *Tagln2*^*−/−*^ DCs treated with H/dU-TG2P (10 µM) or dU-TG2P (5–10 µM) were co-incubated with OTII CD4^+^ T cells in the presence or absence of pOVA (323–339), and then, the cells or cultured supernatants were subjected to a conjugates assay (F), a cytokine production assessment (**g**), and a proliferation test (**h**). All data from **e**–**h** represent the mean of three experiments ± SEM. NS, not significant. *P < 0.01

In a previous report, we demonstrated that cell-permeable recombinant transgelin-2 fused with PTD (TG2P) enhanced cytotoxic T cell-mediated anticancer activity through increased LFA-1 avidity [17], suggesting that TG2P can compensate for endogenous transgelin-2 without viral transduction. WT-TG2P was rapidly internalized into the DCs (Fig. 7a). Figure 7b shows a schematic of the secondary structure of transgelin-2 fused with PTD. However, because we expected that natural transgelin-2 could be degraded by the ubiquitin pathway, as predicted by the program UbiSite [33], we substituted the potential ubiquitination site lysine (K)78 with arginine (R) to generate de-ubiquitinated TG2P (dU-TG2P) (Fig. 7b). Interestingly, dU-TG2P (K78R) showed remarkable stability and persisted longer than 24 h in the cytosol of BMDCs (Fig. 7c). Therefore, we next asked whether dU-TG2P could mimic the actions of transgelin-2 in *Tagln2*^*−/−*^ BMDCs. To this end, *Tagln2*^*−/−*^ BMDCs were incubated with dU-TG2P (10 μM) for 2 h, and then, the cells were seeded on Fn-coated plates. After 90 min, the size of the BMDCs was determined by flow cytometry, revealing that dU-TG2P significantly increased the size of *Tagln2*^*−/−*^ BMDCs (Fig. 7d). To rule out a potential effect of LPS contamination and to confirm the specificity of the recombinant transgelin-2, dU-TG2P was heated inactivated (heat = H, H/dU-TG2P), and its effects on *Tagln2*^*−/−*^ BMDCs were examined. Heat inactivation completely abolished the ability of dU-TG2P to increase the size of *Tagln2*^*−/−*^ BMDCs (Fig. 7d), suggesting that the efficacy of dU-TG2P was solely mediated by transgelin-2. dU-TG2P (10 μM) also significantly increased the spreading of *Tagln2*^*−/−*^ BMDCs on Fn, whereas H/dU-TG2P had a little effect as determined by confocal microscopy and image analysis (Fig. 7e). Consistently, dU-TG2P remarkably increased T cell adhesion to *Tagln2*^*−/−*^ BMDCs in a dose-dependent fashion (Fig. 7f). Further, dU-TG2P-treated *Tagln2*^*−/−*^ BMDCs supported more rapid antigen-specific (OT-II) T cell proliferation and cytokine production than untreated or H/dU-TG2P-treated *Tagln2*^*−/−*^ BMDCs (Fig. 7g, h).

### dU-TG2P potentiates DC-based cancer immunotherapy

**Fig. 8:**
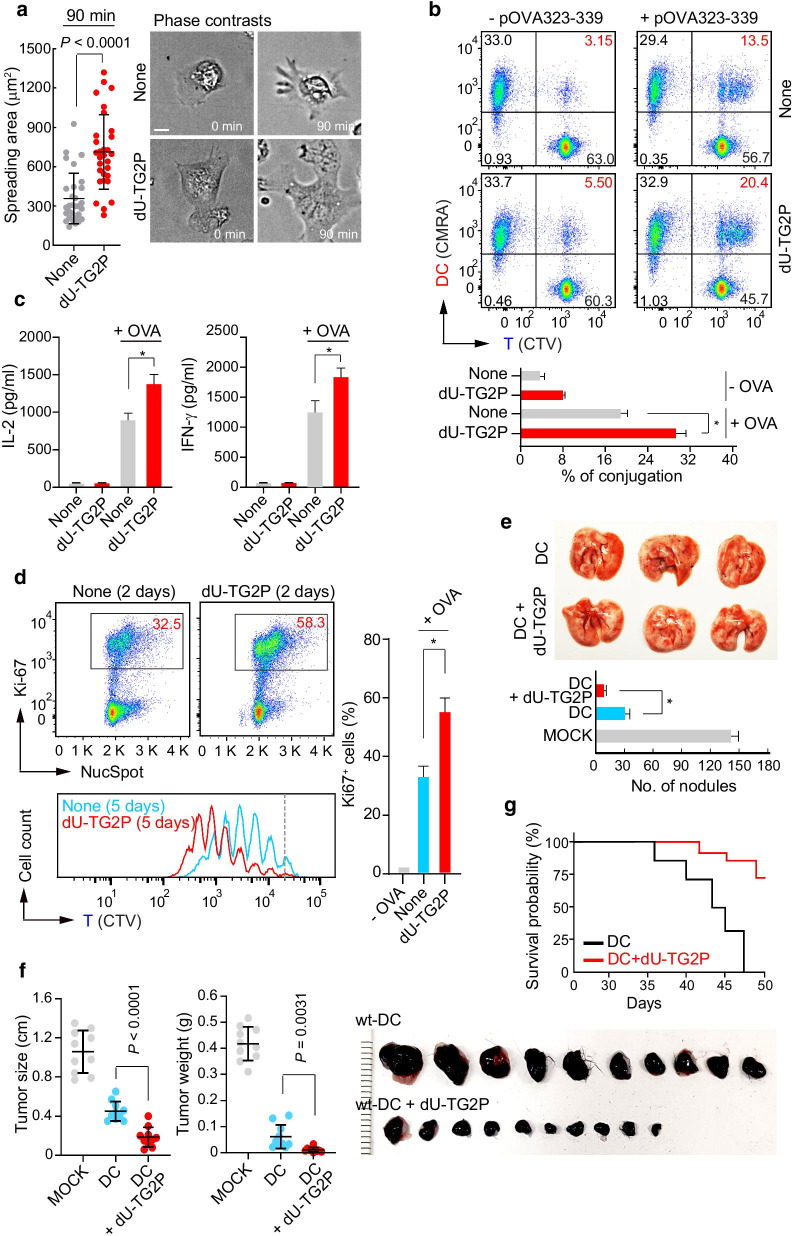
Recombinant dU-TG2P potentiated DC-mediated tumor therapy. **a** dU-TG2P enhances DC cell spreading on Fn. WT DCs treated with dU-TG2P (10 µM) were placed on Fn-coated plates, and the spreading area was measured using ImageJ. Scale bar, 10 μm. **b**–**d** OTII CD4^+^ T cells were co-incubated with nothing or with dU-TG2P-treated WT DCs pulsed with pOVA (323–339), and then, the cells or cultured supernatants were subjected to a conjugate assay (**b**), cytokine production assessment (**c**), and proliferation test (**d**). **e**, **f** Gross images of OVA^+^B16F10 lung and solid tumors, respectively. C57BL/6 mice were injected i.v. with media alone, DCs, or dU-TG2P-treated DCs pulsed with pOVA (257–264). After 7 days, the mice were i.v. (**e**) or s.c. (**f**) injected with OVA^+^B16F10 cells. The metastatic nodules (**e**) and tumor sizes and weights (**f**) were measured after 8 days of OVA^+^B16F10 injection. **g** The survival rates of tumor-bearing mice post-implantation are shown. All data represent the mean of three experiments ± SEM. *P < 0.01

Although we have previously shown that TG2P could enhance cytotoxic T cell-mediated anticancer activity [17], its efficacy may be limited unless a sufficient number of tumor-specific T cell clones are obtained. From this point of view, the amplification of the DC functions may be more important because they can generate numerous T cell clones targeting tumor antigens. We therefore asked whether dU-TG2P could promote the efficacy of WT BMDCs, which would prove useful for DC-based therapeutic applications. WT BMDCs treated with dU-TG2P exhibited significantly increased spreading on Fn (Fig. 8a). Moreover, dU-TG2P treatment produced increased adhesion between WT BMDCs and activated T cells (Fig. 8b), which stimulated the release of increased levels of T cell cytokines (Fig. 8c). Likewise, a larger number of T cells proliferated with co-cultured dU-TG2P-treated BMDCs than with untreated BMDCs (Fig. 8d).

We further asked whether dU-TG2P also could promote the efficacy of WT cDC1s. Similar to the result of WT BMDCs, dU-TG2P increased conjugation between CD8^+^ OTI T cells and WT cDC1s (Additional file 1: Fig. S2A). In addition, increased conjugation correlated with the increased cytokine productions (Additional file 1: Fig. S2B). Accordingly, a larger number of T cells proliferated with co-cultured dU-TG2P-treated cDC1s than with untreated cDC1s (Additional file 1: Fig. S2C).

To evaluate the effects of dU-TG2P-treated BMDCs in the in vivo tumor model, OVA257–264-pulsed untreated BMDCs or dU-TG2P-treated BMDCs were i.v. injected into mice, followed by OVA^+^B16F10 melanoma cells, as described in Fig. 1g. A significant reduction in metastatic nodules was observed in mice with adoptively transferred OVA257–264-pulsed dU-TG2P-treated BMDCs compared to OVA257–264-pulsed untreated BMDCs (Fig. 8e). Consistent with the metastatic model, dU-TG2P-treated BMDCs significantly suppressed tumor growth in mice (Fig. 8f). Kaplan–Meier survival studies showed that mice injected with OVA257–264-pulsed BMDCs had a median survival time of 43 days and mice adoptively transferred with OVA257–264-pulsed dU-TG2P-treated BMDCs had an 80% probability of surviving longer than 47 days (Fig. 8g). Collectively, these results indicate that dU-TG2P is a promising therapeutic approach for DC-based cancer immunotherapy.

## Discussion

Adaptive cellular immunity is initiated by the presentation of a foreign antigen by DCs to antigen-specific naive T lymphocytes [22]. In the periphery upon pathogen encounter, immature DCs uptake antigen and proceed through a maturation process during their migration to the draining LNs. All aspects of immature and mature DC functions rely on dynamic rearrangements of the actin cytoskeleton, which are regulated by various actin-binding proteins and signaling pathways [34]. Despite the importance of DC migration from the periphery to the draining LNs, the roles of the numerous actin regulatory molecules that control this process are incompletely understood. In this study, we showed that transgelin-2 is a critical actin-binding protein that supports the migration of DCs to the draining LNs and DC-dependent priming of T cells for clonal proliferation, which are important functions for the host defense against foreign invaders and neoplastic diseases. Interestingly, recombinant transgelin-2 protein, engineered for cell-penetration and de-ubiquitination, significantly improved the therapeutic activity of WT BMDCs in controlling tumor growth and metastasis in mice.

We previously found that transgelin-2 expression increases in macrophages in response to LPS stimulation [19]. Among three transgelin family members, transgelin-2 is the only isoform that contains an NF-κB consensus motif in the 5′ promoter region and is expressed in immune cells [19], suggesting that this small protein plays a central role in host defenses against infections and neoplastic diseases. We demonstrated that the actin–transgelin-2–LFA-1 axis in cytotoxic CD8^+^ T cells is effective in potentiating adoptive T cell therapy in cases where cancer cells express ICAM-1 on their surface [17]. Indeed, LFA-1 is an essential initiator for the formation of the IS between cytotoxic T cells and cancer cells, and it mediates the polarization of cytotoxic granules toward target cells via tight adhesion to the target cells [35]. However, not all cancer cells express ICAM-1 [17]. Moreover, some reports have demonstrated that the expression of ICAM-1 is positively correlated with a more aggressive tumor phenotype and metastatic potential [36, 37]. By contrast, the antitumor functions of DCs are mediated through the initiation of various adaptive immune mechanisms, including clonal expansion of antigen-specific CD4 and CD8 T cells. Thus, improving DC functions represents a more attractive strategy than directly enhancing T cell functions. In this respect, cell-permeable peptides that promote transgelin-2-like functions in DCs have a potential clinical value as a cancer immunotherapy based on DCs.

In some cancer cells, transgelin-2 is known to inhibit cellular motility by suppressing actin polymerization [38]. Consistently, transgelin-2 was found to be more downregulated in metastatic tumors than in primary cancers [38]. However, as observed in this study, the reduced migration of *Tagln2*^*−/−*^ BMDCs toward chemokine gradients or into the draining LN unambiguously suggests that transgelin-2 is involved in the dynamic movement of DCs. This conclusion is also corroborated by our previous works, in which transgelin-2- KO in T cells or macrophages reduced their motility [16, 19]. Dynamic actin regulation by transgelin-2 appears to be mediated by its ability to induce small filopodia-like protrusions at the leading edge of migrating cells and to control podosome formation [27]. Filopodia and podosomes are important subcellular architectures that sense the external environment and degrade the ECM during DC migration, respectively [27]. Moreover, the fact that *Tagln2*^*−/−*^ BMDCs showed a remarkable decrease in F-actin levels suggests that transgelin-2 is involved in actin polymerization in vivo [16]. We believe that the reduced F-actin content in transgelin-2 KO cells is due to the rapid decomposition of polymerized F-actin as this protein directly stabilizes F-actin structures after polymerization but does not increase actin polymerization [16, 18, 39]. In cancer cells, however, these characteristics of transgelin-2 may be involved in the process of tumorization in a wide range of cancers [14, 40]. In this respect, transgelin-2 may be a promising target protein for cancer therapy. In fact, several reports using chemical compounds or microRNAs targeting the *Tagln2* gene have shown potential positive results in the suppression of cancer development and metastasis. These interesting features of transgelin-2 suggest that this small actin-binding protein acts as a double-edged sword in the context of cancer and immune cells.

One interesting lingering question is the mechanism by which transgelin-2 in BMDCs mediates increased T cell adhesion, thereby enhancing T cell clonal proliferation. One possibility is that transgelin-2 may participate in the growth of small microvilli on the DC surface, and these multiple finger-shaped structures could provide a physical means of clustering adhesion molecules to support T cell adhesion. Interestingly, a previous report demonstrated that DCs can produce multifocal synapses with clustered T cells via microvilli [41]. These microvilli on the DCs exhibited a high density of antigen-presenting molecules and co-stimulatory molecules, providing the physical basis for the preferential adhesion of both CD4^+^ and CD8^+^ T cells [41, 42]. Along these lines, Jung et al. and our group recently found that T cell microvilli also provide a platform to cluster important T cell molecules, including TCR, TCR complex, co-receptors, and co-stimulatory molecules [43, 44], suggesting that initial recognition and adhesion are mediated through polarized microvilli between DCs and T cells.

DCs are the most potent antigen-presenting cell type and are key players in tumor-specific immune responses. This characteristic has been exploited by DC therapy, in which DCs are loaded with tumor-associated antigens and applied to patients to induce immune responses against tumor antigens. However, although multiple clinical trials have been performed, clinical scores have been largely disappointing. This is due in part to insufficient antigen presentation and T cell activation, migratory potential, and cytokine production [45]. In this regard, accumulating evidence suggests cDC1s—which are different from monocyte-derived DCs—play an integral role in tumor immunity and are a good candidate for vaccination purposes [45]. In the present study, we found that transgelin-2 is also induced in Flt3 ligand-induced cDC1s. Moreover, reduction of tumor growth control by *Tagln2*^*−/−*^ cDC1s strongly suggests that transgelin-2 is an important actin regulator for optimal action of various DC subsets. Therefore, it will be very interesting to investigate the global gene signatures of WT BMDCs and *Tagln2*^*−/−*^ BMDCs and to compare these with cDC1s. Further, it will be interesting to test whether cell-permeable transgelin-2 can change the gene signatures of BMDCs toward the cDC1s.

Abnormal changes in the actin cytoskeleton contribute to the growth, metastasis, and invasion of cancer cells. However, because the actin cytoskeleton is indispensable for all living cells, drugs that target the actin cytoskeleton of tumor cells may exhibit off-target toxicity in noncancerous cells. To overcome this matter, targeting actin-regulating factors with altered expression in cancers may become an alternate therapy to increase tumor toxicity. Interestingly, transgelin-2 is essential for both cancer development and immune functions [23, 40]. This suggests that transgelin-2 can act as a double-edged sword depending on how we apply this protein to cancer therapy. Transgelin-2 plays an important role in fine-tuning the structure and function of the actin cytoskeleton, which is crucial for DC migration, antigen presentation, and the formation of the immune synapse between DCs and T cells. Engineering and clinical application of this protein may unveil a new era in DC-based cancer immunotherapy.

## Conclusion

Adaptive cellular immunity is initiated by a series of actions of DCs that uptake and present foreign antigens, migrate to the draining LNs, and interact with antigen-recognizing T cells. Transgelin-2, a 22 kDa actin-binding protein, is upregulated in DCs during maturation and LPS activation. *Tagln2*^*−/−*^ DCs exhibited significant defects in their abilities to home to draining LNs and to form optimal contacts with cognate CD4^+^ T cells to prime T cells, and these changes were associated with a failure to suppress tumor growth and metastasis of B16F10 melanoma cells in mice. Recombinant transgelin-2 protein, engineered for cell-penetration and de-ubiquitination, potentiated DC functions to suppress tumor growth and metastasis, demonstrating that this small-actin binding protein represents a promising therapeutic approach for DC-based cancer immunotherapy.

## Materials and methods

### Antibodies and reagents

Rabbit polyclonal anti-transgelin-2 antibody was raised in rabbits using purified full-length transgelin-2 (AbFrontier, Seoul, Korea). In addition, the following antibodies were used: goat polyclonal anti-TAGLN1 (Santa Cruz Biotechnology, Dallas, TX, USA); rabbit polyclonal anti-β-actin; rabbit polyclonal antibodies against p-PI3K, t-PI3K, p-AKT, t-AKT, p-p38, t-p38, p-ERK, t-ERK, His, HRP-conjugated anti-mouse IgG, anti-goat IgG, and anti-rabbit IgG (Cell Signaling Technology, Danvers, MA, USA); mouse monoclonal anti-TAGLN3 and anti-vinculin (Abcam, Cambridge, MA, USA); and antibodies for FITC-conjugated CD40 (MA5-16506), MHCII (11-5322-82), CD18 (LFA-1β; 11-0181-82), ICAM-1 (11-0541-82), CD11c (17-0114-82), PE-conjugated CD86 (12-0862-82), CD80 (12-0801-82), CD25 (120251-82), CD69 (12-0691-82), CXCR4 (12-9991-82), CCR7 (12-1971-82), APC-conjugated CD11c (17-0114-82), and B220 (17-0452-82) (eBioscience, San Diego, CA, USA); FITC-conjugated CD24 (M1/69), CD103 (2E7), and SIRPα (P84) (BioLegend, San Diego, CA, USA). All antibodies for flow cytometry were used at a dilution of 1:100. Phalloidin-TRITC, lipopolysaccharide, and Fn were purchased from Sigma Aldrich (St. Louis, MO, USA). GM-CSF, CCL-3, CCL-5, CCL-19, CCL-21, and SDF-1α were purchased from Peprotech Inc. (Rocky Hill, NJ). Alexa647-phalloidin, CellTracker CMFDA-green, CMRA-Orange dyes, anti-mouse Alexa 647, and anti-rabbit Alexa488 were purchased from Invitrogen (Carlsbad, CA, USA). A CellTrace™ Violet (CTV) Cell Proliferation Kit was purchased from Thermo Fisher Scientific (Waltham, MA, USA). OVA peptide fragments (323–339 and 257–264) were purchased from GeneScript (San Francisco, CA, USA). Flt3L-Ig was purchased from Bio-X-Cell (West Lebanon, NH, USA). CD45R (B220) MicroBeads was purchased from Miltenyi Biotec. (Bergisch Gladbach, Germany).

### Cells

B16F10 (CRL-6475) cell lines were purchased from ATCC. A stable B16F10 cell line expressing membrane-bound OVA (OVA^+^B16F10) was produced by transient transfection with pCL-neo-mOVA (Addgene, Cambridge, MA) using Lipofectamine 2000 reagent (Invitrogen) and selection with G418 (InvivoGen, San Diego, CA, USA). For BMDCs cultures, 5 × 10^6^ BM cells were cultured in 10 mL of RPMI supplemented with 20 ng/mL recombinant murine GM-CSF for 7 to 9 days. GM-CSF was added every 3 days. To generate cDC1s, 3 × 10^6^ BM cells were incubated in 3 mL of RPMI supplemented with 200 ng/ mL Flt3-L for 9 days. Flt3-L was added every 2 days and cDC1s (CD11c^+^B220^−^) were isolated by anti-B220 positive selection beads to exclude plasmacytoid DCs (CD11C^+^B220^+^) for further experiments. However, unless otherwise indicated (for Additional file 1: Figs. S1 and 2), we used GM-CSF-induced BMDCs for most of the experiments. Naive CD4^+^ T cells were purified from the mouse spleen and LNs by negative selection using an EasySep magnetic separation system (Stemcell Technologies, Vancouver, Canada). To generate mouse T cell blasts, OTII CD4^+^ T cells were incubated in 2 µg/mL anti-CD3/28-coated culture plates with 100 U/mL rIL-2 for 48 h and cultured further for 3 days with 100 U/mL rIL-2.

### Mice

C57BL/6 wi mice and *OTII TCR* transgenic mice (C57BL/6 background) were purchased from Damul Science (Korea) and Jackson Laboratories (Bar Harbor, ME, USA), respectively. All mice were housed under specific pathogen-free conditions. Transgelin-2 (*Tagln2*^−/−^) KO mice have been described previously [16]. All experimental methods and protocols were approved by the Institutional Animal Care and Use Committee of the School of Life Sciences, Gwangju Institute of Science and Technology, and carried out in accordance with their approved guidelines (IACUC GIST-2015–04).

### Western blotting

To analyze transgelin family expression in DCs, BM cells were harvested at the indicated day during differentiation with GM-CSF and Flt3L, respectively, and cells were lysed in ice-cold lysis buffer (50 mM Tris–HCl, pH 7.4, containing 150 mM NaCl, 1% Triton X-100, and one tablet of complete protease inhibitors) for 15 min on ice. Cell lysates were centrifuged at 16,000 × *g* for 30 min at 4 °C, and the supernatants were eluted with sodium dodecyl sulfate (SDS) sample buffer (100 mM Tris–HCl, pH 6.8, 4% SDS, and 20% glycerol with bromophenol blue) and heated for 5 min. The proteins were separated by SDS polyacrylamide gel electrophoresis on 10%–15% gels and were transferred to nitrocellulose membranes using a Trans-Blot SD semidry transfer cell (Bio-Rad, Hercules, CA). The membrane was blocked in 5% skim milk for 1 h, rinsed, and incubated with the appropriate antibodies in TBS containing 0.1% Tween 20 (TBST) and 0.5% skim milk overnight. Excess primary antibody was then removed by washing the membrane three times in TBST. The membrane was then incubated with 0.1 μg/mL peroxidase-conjugated secondary antibodies (anti-rabbit or anti-mouse) for 1 h. After three washes with TBST, bands were visualized using western blotting detection reagents (EZ-Western Lumi Femto Kit; DoGenBio, Seoul, South Korea) and were then exposed to an X-ray film (Kodak, Rochester, NY).

### Analysis of differentiation and activation of DCs

WT or *Tagln2*^*−/−*^ BMDCs (1 × 10^6^) were activated with 200 ng/mL of lipopolysaccharide (LPS), harvested, and blocked with a rat anti-mouse CD16/CD32 antibody (mouse Fc Block, BD Pharmingen). Cells were then stained with activation markers, including CD11c, CD80, CD86, CD40, and MHC-II, for flow cytometry. To examine T cell-mediated DC activation in vitro, 1 μg/mL of pOVA (323–339)-pulsed WT or *Tagln2*^*−/−*^ BMDCs (1 × 10^5^) was co-cultured with OTII CD4^+^ T cells (5 × 10^5^) for 24 h, and the supernatants were subjected to ELISA assay to examine cytokine production.

### Cell spreading on Fn

BMDCs were plated on coverslips coated with or without 10 μg/mL of Fn for 90 min. The cells were fixed for 10 min with 4% paraformaldehyde and permeabilized with 0.1% Triton-X (Sigma-Aldrich) in PBS for 10 min at room temperature (RT). The coverslips were then incubated with TRITC-conjugated phalloidin at RT for 30 min, washed, mounted onto slide glass using Vectashield (VectorLabs, Burlingame, CA), and imaged using a FV-1000 confocal microscope (Olympus, Tokyo, Japan) To measure cell spreading area, the captured images were analyzed using ImageJ software (NIH) as follows: threshold values were set to define the cell edge, and a mask was then created for each cell to get the total cell area (with arbitrary units) within the mask. Cell size was determined by flow cytometry after detachment of the BMDCs with 10% EDTA.

### Conjugation assay

OTII CD4^+^ or OTI CD8^+^ T cells were stained with Cell Tracker Green CMFDA, and WT or *Tagln2*^*−/−*^ BMDCs or Flt3L-induced cDC1s were stained with Cell Tracker Orange CMRA for 30 min. The cells were then washed and resuspended in RPMI 1640 media. For conjugation, DCs were incubated with T cells (1:5 ratio) for 2 h in the presence or absence of pOVA (323–339, 1 μg/mL) or pOVA (257–264, 1 μg/mL). The relative proportion of green, orange, and green and orange-positive events in each tube was determined by FACS Canto (BD Biosciences, San Jose, CA) and analyzed with FlowJo software (Treestar, San Carlos, CA). The number of gated events counted per sample was at least 10,000. The percentage of conjugated T cells was determined as the number of dual-labeled (green and orange-positive) events divided by the number of green-positive T cells.

### Determination of in vitro and in vivo T-cell activation

To examine in vitro T-cell activation, the indicated concentration of pOVA (323–339, 10^−3^–10^1^ μg/mL)-pulsed WT or *Tagln2*^*−/−*^ BMDCs (1 × 10^5^) was co-cultured with OTII CD4^+^ T cells (5 × 10^5^) for 24 h, and the supernatants were subjected to ELISA assay to determine cytokine secretion. In addition, the cells were stained with anti-CD69 or CD25 to determine DC activation. To determine the effects of dU-TG2P, BMDCs or Flt3L-induced cDC1s (1 × 10^5^) were treated with dU-TG2P along with pOVA (323–339, 1 μg/mL) or pOVA (257–265, 1 μg/mL) for 2 h and co-cultured with OTII CD4^+^ T cells (5 × 10^5^) or OTI CD8^+^ T cells (5 × 10^5^) for 24 h, and the supernatants were subjected to ELISA assay to determine cytokine secretion. For in vivo T-cell priming, WT or *Tagln2*^*−/−*^ BMDCs (3 × 10^6^) were pulsed with pOVA (323–339, 1 μg/mL) for 2 h and injected s.c. into the footpads of OTII mice, and the cells were isolated from the popliteal lymph node at 24 h post-injection. In general, to stain the cells with fluorescently conjugated antibodies, the cells were blocked with anti-FcγR antibody (CD16/32, clone 2.4G2) and then stained for surface activation markers. Data were acquired using a FACS Canto and analyzed with FlowJo software.

### In vitro migration assay using a Transwell system

Transwell cell migration was assayed using a 96-well Boyden chamber (ChemoTx plate, Neuroprobe, Inc., Gaithersburg, MD) according to the manufacturer’s instructions. The Boyden chamber was assembled with polyvinylpyrrolidone-free polycarbonate filters (3–5-µm pore size). WT or *Tagln2*^*−/−*^ BMDCs (1 × 10^6^) were added to the Fn-coated upper compartment, and media containing 200 ng/mL of each chemokine were added to the lower compartment. The apparatus was incubated for 4 h at 37 °C in a humidified CO_2_ incubator. Cells on the bottom wells were harvested and resuspended in 300 µL of PBS, and the number of cells was counted using a FACS Canto for a fixed period of time (300 s) under constant middle pressure.

### In vivo migration assay

To evaluate DC migration, a mixture of 2 × 10^6^ WT (CMFDA-green) and 2 × 10^6^ *Tagln2*^*−/−*^ BMDCs (CMRA-orange) was injected into the footpads of WT recipient mice. A popliteal lymph node was harvested at 24 h post-injection. The popliteal LNs were fixed in 4% paraformaldehyde in PBS at 4 °C overnight. On the next day, the samples were washed and incubated in PBS with 30% sucrose (w/v) (Sigma-Aldrich) overnight at 4 °C. The samples were then embedded in Tissue-Tek® O.C.T. Compound (Thermo Fisher Scientific) and frozen using 2-methylbutane, cooled with liquid nitrogen. 10 μm sections were cut using a Leica CM1800 cryostat. For immunostaining, tissue sections were blocked for 2 h at RT in 10% normal goat serum (Sigma-Aldrich). The sections were incubated with fluorescently conjugated anti-B220 antibody at RT for 30 min in 10% goat normal serum. The samples were washed three times to remove unbound antibody and mounted in Permount solution (Thermo Fisher Scientific). Images were acquired with a confocal microscope and analyzed with Fluoview software. To quantify the number of migratory DCs, single-cell suspensions from the draining popliteal LNs were obtained by digestion in collagenase D, and the % of migrating DCs was quantified using FACS Canto. In some experiments, the excised lymph node was photographed for size determination. Draining popliteal LNs were harvested from the left hind limb, which were injected with cells through the footpad, whereas nondraining LNs were excised from the right hind limb.

### Immunocytochemistry

To analyze the podosome dynamics, BMDCs were harvested and seeded on poly-L-lysine-coated glass coverslips in a 12-well plate (2 × 10^6^ cells/well in supplemented medium) and incubated for the indicated time in the presence of 200 ng/mL of LPS at 37 °C and 5% CO_2_. To analyze the localization of transgelin-2, BMDCs were incubated for 24 h in the same condition. Coverslips were washed once with warmed PBS, followed by fixation with 4% paraformaldehyde in PBS at RT for 10 min. After permeabilization using 0.1% Triton X-100, the cells were stained with 10 µg of anti-vinculin and anti-transgelin-2 antibodies at 4 °C overnight. The next day, the coverslips were washed with PBS two times, and the cells were stained with mouse anti-Alexa647 and rabbit anti-rabbit Alexa488 secondary antibodies. For actin staining, permeabilized cells were incubated with anti-Alexa 647- or TRITC-conjugated phalloidin (1:100) for 1 h at RT. The coverslips were mounted onto slideglass using Vectashield (Vector Labs) and imaged using a confocal microscope. For quantitation of cells with podosomes, the cells were assessed for the presence of at least one clearly identifiable podosome with an F-actin-rich core. At least 100 cells were scored per sample, with a minimum of three biological replicates.

### DC–T cell interactions via intravital two-photon microscopy

For in vivo imaging, pOVA (323–339, 1 µg/mL)-pulsed WT or *Tagln2*^*−/−*^ BMDCs (3 × 10^6^) were stained with Cell Tracker CMFDA-green and injected s.c. into the footpads of WT recipient mice. After 24 h, purified OTII CD4^+^ T cells were stained with CMRA-orange and adoptively transferred to recipient mice intravenously. Mice were anesthetized with isoflurane, and the popliteal LNs were surgically exposed. Imaging was performed on a Zeiss LSM 880 microscope equipped with a MaiTai laser (Coherent) tuned to 750 nm in combination with an NDD2 BIG2 GaAsP detector and a 20 × water-dipping lens (NA 1.0, Zeiss) using ZEN v2.1 acquisition software. Images were collected with a typical voxel size of 0.593 × 0.593 × 1.0 μm and a volume dimension of 607.28 × 607.28 × 200 μm to create a three-dimensional data set. For four-dimensional data sets, images were collected with a typical voxel size of 0.45 × 0.45 × 1.5 μm and a volume dimension of 425.1 × 425.1 × 30 μm. This volume collection was repeated every 60 s for up to 2 h. To analyze the number of DC-contacted T cells and the speed, displacement, and duration of these interactions, tracks were generated for T cells and analyzed using Imaris software. Data were plotted using Prism (GraphPad).

### Time-lapse video microscopy

For the dynamic analysis of DC spreading and protrusion, time-lapse imaging was conducted on an EVOS system (EVOS™ FL Digital Inverted Fluorescence Microscope, Fisher Scientific, Paisley, Scotland, UK). WT or *Tagln2*^*−/−*^ BMDCs (3 × 10^5^) were seeded on 10 µg/mL of Fn-coated 12-well non-tissue culture plates for 10 min at 37 °C, and the plates were immediately placed in the chamber of the EVOS unit (which was programmed to supply 5% CO_2_ and maintained at 37 °C constant temperature). The cells were recorded in the presence or absence of 200 ng/mL CCL19 every 10 s for up to 1 h. Sequential images were analyzed using ImageJ software.

### Scanning electron microscopy

For scanning EM, cells were fixed with 2.5% glutaraldehyde solution for 2 h, rinsed with PBS for 5 min, and fixed in OsO_4_ for 2 h. The samples were then dehydrated through incubation with a graded ethanol series over 30 min and dried in a critical point dryer. The samples were prepared by sputter coating with 1–2 nm gold–palladium and analyzed using FE-SEM (HITACHI, Tokyo, Japan).

### B16F10 melanoma tumor model and isolation of TILs

To evaluate transgelin-2 functions in T cells for tumor suppression (for Fig. 1b), PBS (none), WT (OTI-T), KO (*Tagln2*^*−/−*^ OTI-T), or transgelin-2-overexpressing T (TG2OE OTI-T) cells were adoptively transferred into tumor-bearing C57BL/6 mice at days 7, 10, and 13 post-tumor implantation. All mice were sacrificed at day 25, and tumors were isolated and weighed.

To evaluate the effect of *Tagln2*^*−/−*^ in mice (for Fig. 1c), OVA^+^B16F10 cells (3 × 10^5^) were s.c. injected into the dorsal flank region of age- and sex-matched WT or *Tagln2*^*−/−*^ mice. To evaluate transgelin-2 functions in DCs for tumor suppression, BMDCs (1 × 10^7^) from WT or *Tagln2*^*−/−*^ were pulsed with 1 µg/mL of pOVA for 2 h and i.v. injected into 8-week-old WT mice. At day 7, OVA^+^B16F10 cells (3 × 10^5^) were s.c. or i.v. injected into the dorsal flank region to induce solid and metastatic tumor models, respectively. Mice were sacrificed at day 8 post-inoculation with tumor cells. At the end of the experiments, tumors were isolated, weighed, and photographed for gross morphology. To analyze TILs, tumor tissues were dissected and mechanically disaggregated before digestion with collagenase D (1 mg/mL, Roche) for 30 min at 37 °C. After digestion, all of the cells were passed through 70-µm filters, and leukocytes were isolated by centrifugation using 38% Percoll for 30 min. Pellets were resuspended with PBS, stained with anti-CD3 and CD8 antibodies, and analyzed by flow cytometry. To analyze cytokine production, isolated TILs were stimulated with PMA/Ionomycin (200 nM/ 1 µM) in the presence of Brefeldin A (1 µg/mL) for 4 h at 37 °C. Cells were subsequently collected and stained for CD8 followed by fixation with IC fixation Buffer (eBioscience) for 20 to 30 min at RT. Then, the cells were washed twice with 1 × Permeabilization Buffer (eBioscience) and stained with IFN-γ antibody. After washing, cells were analyzed by flow cytometry.

### Generation of OVA-specific T cells and ex vivo proliferation assay

To measure proliferation and cytokine production from OVA-specific T cells, DCs from WT or *Tagln2*^*−/−*^ (1 × 10^7^) were pulsed with 1 µg/mL of pOVA (257–265) for 2 h and i.v. injected into 8-week-old C57BL/6 WT mice. At day 7, CD3^+^ T cells were isolated from the LNs and spleens, stained with CTV, and co-cultured with 1 µg/mL of pOVA (257–265)-pulsed WT BMDCs for 4 days for clonal expansion. The proliferative CTV-positive cells that were in a live cell gate were quantified by flow cytometry. To measure the IL-2 or IFN-γ cytokines from OVA-specific T cells, co-cultured supernatants were harvested at 24 h and subjected to ELISA.

### Generation of dU-TG2P mutant and purification of recombinant TG2P or dU-TG2P

Recombinant transgelin-2 protein fused with PTD (TG2P) was described previously [17]. To generate a de-ubiquitinated mutant of TG2P, a potential ubiquitinated amino acid residue was predicted using the program UbiSite [33], and the resulting residue, K78, was mutated to an R by site-directed mutagenesis. The resulting PCR-amplified cDNAs encoding dU-TG2P were ligated into the pET-28a vector (Novagen, Madison, WI). Expression of recombinant TG2P and dU-TG2P in *Escherichia coli* BL21 (DE3) cells has been described previously [17].

### Introduction of TG2P (dU-TG2P) into DCs

DCs were washed and incubated with 10 μM of TG2P or dU-TG2P for 2 h at 37 °C in serum-free media. The cells were then washed with PBS and resuspended in media for further assays. To rule out the potential for LPS contamination, dU-TG2P was heat-inactivated by boiling for 5 min.

### Proliferation assay

OTII CD4^+^ or OTI CD8^+^ T cells were stained with CTV and co-cultured with 1 µg/mL of pOVA (323–339)-pulsed BMDCs or pOVA (257–264)-pulsed cDC1s (1 × 10^5^) for 2, 3 or 5 days. Total cells were fixed with Cell Fixation & Cell Permeabilization Kit (Thermo Fisher Scientific) for 1 h at 4 °C and stained with anti-Ki67 along with 1 μL of NucSpot Far-Red for 1 h at 4 °C in the dark. The samples were washed with PBS and analyzed by flow cytometry. The percent of proliferative populations was acquired from the gate in a CTV-positive population.

### Statistics

Student’s t-test and one-way ANOVA analysis of variance (corrected for all pairwise comparisons) were performed using Prism software. *P*-values < 0.05 were considered statistically significant.

## Supplementary Information

**Additional file 1: Fig. 1.** Tagln2^−/−^ cDC1s do not optimally control B16F10 tumor growth in mice. (A) Flt3L-induced cDCs were generated from WT or *Tagln2*^−/−^ BM cells, and the expressions of the indicated surface markers were analyzed by flow cytometer. (B) Expression of transgelin-2 in Flt3L-induced cDC1s. (C) Gross images of OVA+B16F10 solid tumors. C57BL/6 mice were injected i.v. with media alone, WT cDC1s, or *Tagln2*^*−/−*^ cDC1s. After 7 days, the mice were s.c. injected with OVA+B16F10 cells. The tumor weights and sizes were quantified at day 8 post tumor inoculation. All data represent the mean of three experiments ± SEM. *P < 0.01. **Fig. 2.** Recombinant dU-TG2P potentiated cDC1-mediated tumor therapy. (A) OTII CD8^+^ T cells were co-incubated with none- or dU-TG2P-treated pOVA (257–264)-pulsed cDC1s. After 2 h, the cells were then subjected for conjugation assay. (B) After 24 h, culture supernatants from (A) were subjected for cytokine production. (C) After 3 days, T cell proliferation was assessed by Ki-67/NucSpot double-positive populations (top) and CTV dilution (bottom). All data represent the mean of three experiments ± SEM. *P < 0.01 (PDF 7390 KB)

**Additional file 2.** Time-lapse video showing membrane extensions and cell body movements of WT DCs on Fn-coated plates in the absence of CCL19 (MP4 2462 KB)

**Additional file 3.** Time-lapse video showing membrane extensions and cell body movements of *Tagln2*^−/−^ DCs on Fn-coated plates in the absence of CCL19 (MP4 1233 KB)

**Additional file 4.** Time-lapse video showing cell spreading, membrane extensions, and cell body movements of WT DCs on Fn-coated plates in the presence of CCL19 (MP4 2539 KB)

**Additional file 5.** Time-lapse video showing cell spreading, membrane extensions, and cell body movements of *Tagln2*^−/−^ DCs on Fn-coated plates in the presence of CCL19 (MP4 1032 KB)

## Data Availability

All data generated or analyzed during this study are included in this published article [and its additional files].

## Abbreviations

BM: Bone marrow
BMDCs: Bone marrow-derived DCs
cDC1s: Conventional type 1 DCs
CTV: CellTrace™ Violet
DC: Dendritic cell
dU-TG2P: De-ubiquitinated TG2P
ECM: Extracellular matrix
EV: Empty vector
Flt3L: FMS-like tyrosine kinase 3 ligand
Fn: Fibronectin
GM-CSF: Granulocyte–macrophage colony-stimulating factor
KO: Knockout
ICAM-1: Intercellular adhesion molecule-1
IS: Immunological synapse
I.V.: Intravenously
LPS: Lipopolysaccharide
LFA-1: Lymphocyte function-associated antigen-1
LNs: Lymph nodes
OVA: Ovalbumin
PTD: Protein transduction domain
SDS: Sodium dodecyl sulfate
S.C.: Subcutaneously
SEM: Scanning electron microscopy
TBST: TBS containing 0.1% Tween 20
TG2: Transgelin-2
TG2P: Transgelin-2 fused with PTD
TILs: Tumor-infiltrating lymphocytes
WT: Wild-type
