# Supplementary material for: Cell-permeable transgelin-2 as a potent therapeutic for dendritic cell-based cancer immunotherapy
Source: J Hematol Oncol. 2021 Mar 17;14:43. doi: 10.1186/s13045-021-01058-6 (PMC7968273; doi:10.1186/s13045-021-01058-6)

Figure S1. *Tagln2*-knockout cDCs do not optimally control tumor growth in mice

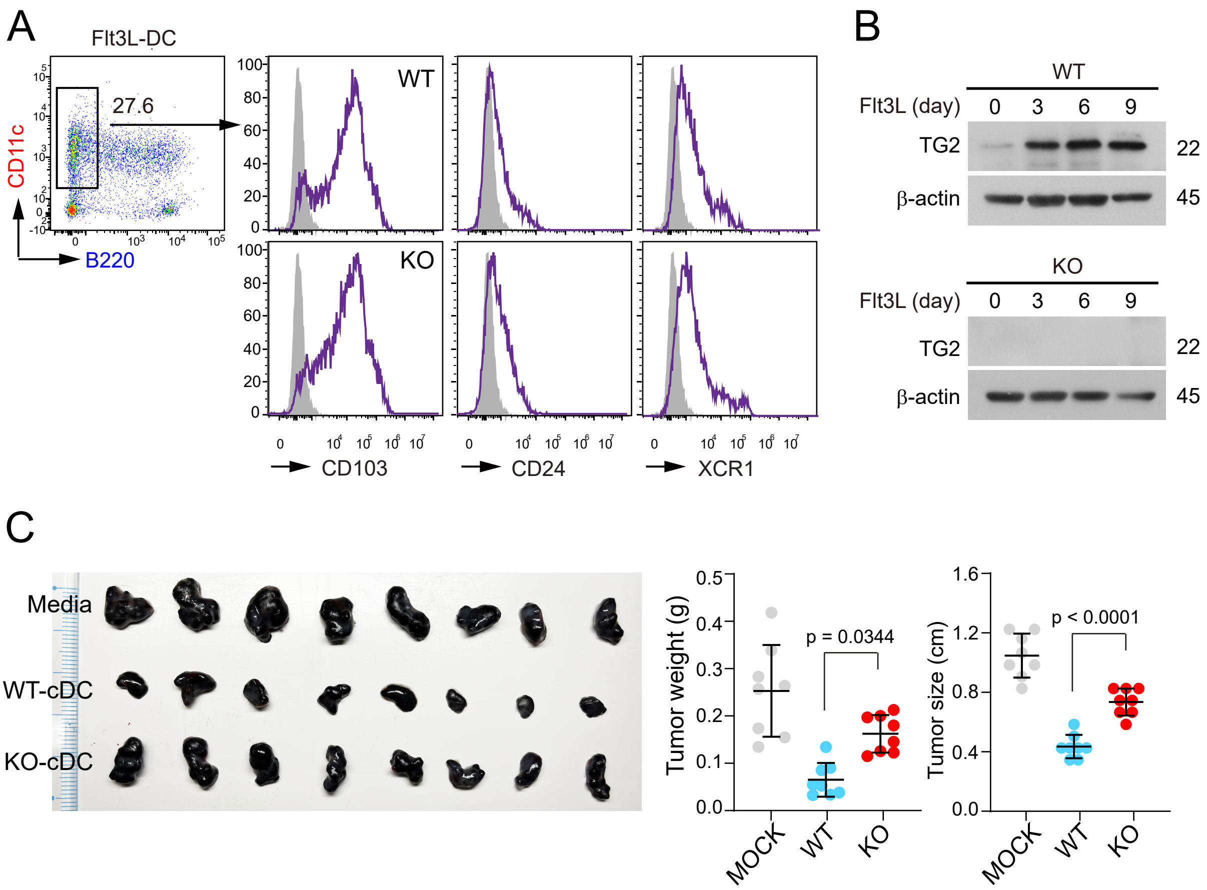

Figure S2. Recombinant dU-TG2P potentiated cDC functions *in vitro*

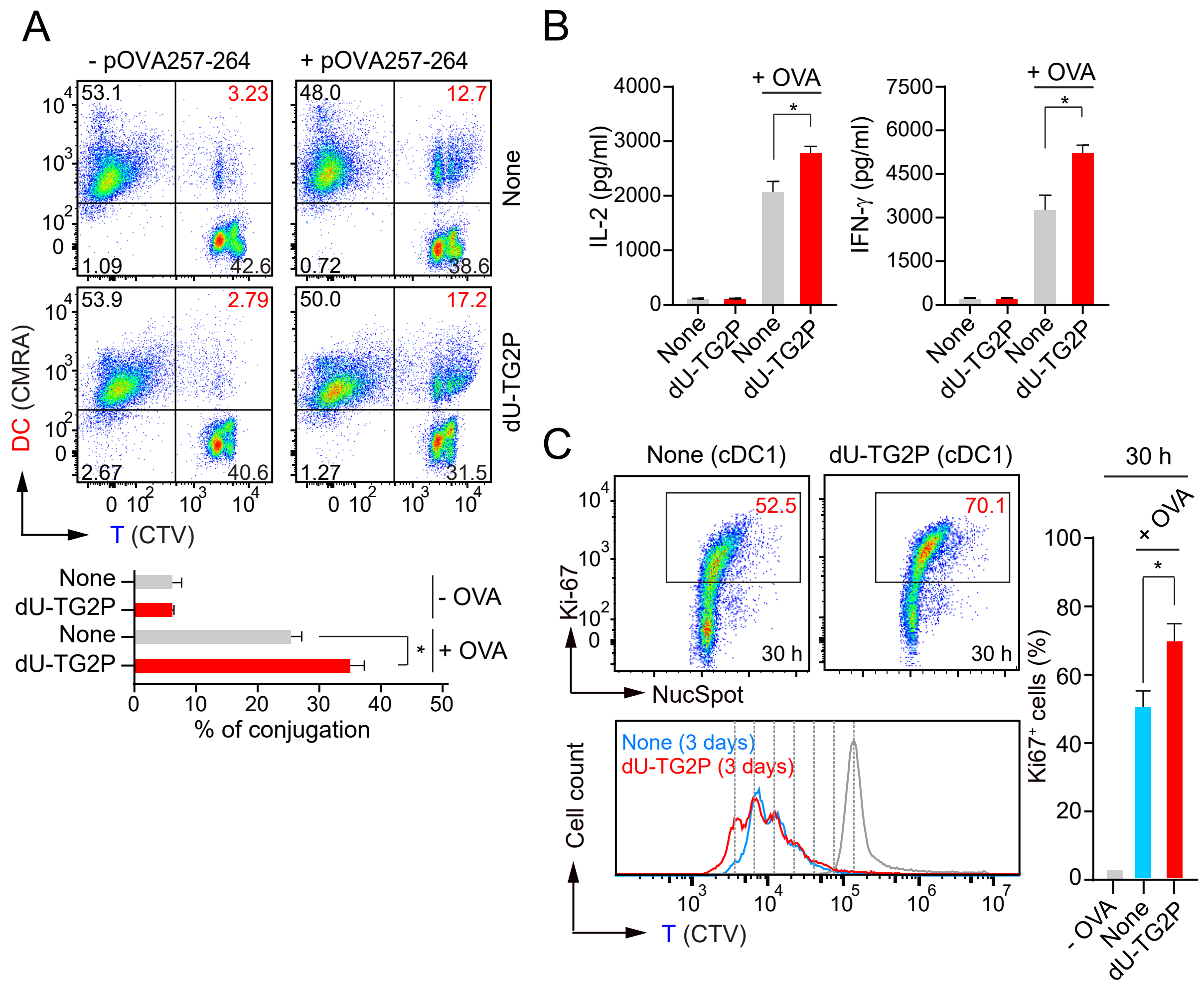

Supplement: Supplementary file 1 — Additional file 1: Fig. 1. Tagln2−/− cDC1s do not optimally control B16F10 tumor growth in mice. (A) Flt3L-induced cDCs were generated from WT or Tagln2−/− BM cells, and the expressions of the indicated surface markers were analyzed by flow cytometer. (B) Expression of transgelin-2 in Flt3L-induced cDC1s. (C) Gross images of OVA+B16F10 solid tumors. C57BL/6 mice were injected i.v. with media alone, WT cDC1s, or Tagln2−/− cDC1s. After 7 days, the mice were s.c. injected with OVA+B16F10 cells. The tumor weights and sizes were quantified at day 8 post tumor inoculation. All data represent the mean of three experiments ± SEM. *P < 0.01. Fig. 2. Recombinant dU-TG2P potentiated cDC1-mediated tumor therapy. (A) OTII CD8+ T cells were co-incubated with none- or dU-TG2P-treated pOVA (257–264)-pulsed cDC1s. After 2 h, the cells were then subjected for conjugation assay. (B) After 24 h, culture supernatants from (A) were subjected for cytokine production. (C) After 3 days, T cell proliferation was assessed by Ki-67/NucSpot double-positive populations (top) and CTV dilution (bottom). All data represent the mean of three experiments ± SEM. *P < 0.01 (PDF 7390 KB) [file 13045_2021_1058_MOESM1_ESM.pdf]
